# Methyltransferase-like protein 7A (METTL7A) promotes cell survival and osteogenic differentiation under metabolic stress

**DOI:** 10.1038/s41420-021-00555-4

**Published:** 2021-06-30

**Authors:** Eugene Lee, Ju-young Kim, Tae-Kyung Kim, Seo-Young Park, Gun-Il Im

**Affiliations:** Research Institute for Integrative Regenerative Biomedical Engineering, Dongguk University, Goyang, 10326 Republic of Korea

**Keywords:** Nutrient signalling, Stem-cell differentiation

## Abstract

While bone has an inherent capacity to heal itself, it is very difficult to reconstitute large bone defects. Regenerative medicine, including stem cell implantation, has been studied as a novel solution to treat these conditions. However, when the local vascularity is impaired, even the transplanted cells undergo rapid necrosis before differentiating into osteoblasts and regenerating bone. Thus, to increase the effectiveness of stem cell transplantation, it is quintessential to improve the viability of the implanted stem cells. In this study, given that the regulation of glucose may hold the key to stem cell survival and osteogenic differentiation, we investigated the molecules that can replace the effect of glucose under ischemic microenvironment of stem cell transplantation in large bone defects. By analyzing differentially expressed genes under glucose-supplemented and glucose-free conditions, we explored markers such as methyltransferase-like protein 7A (METTL7A) that are potentially related to cell survival and osteogenic differentiation. Overexpression of METTL7A gene enhanced the osteogenic differentiation and viability of human bone marrow stem cells (hBMSCs) in glucose-free conditions. When the in vivo effectiveness of METTL7A-transfected cells in bone regeneration was explored in a rat model of critical-size segmental long-bone defect, METTL7A-transfected hBMSCs showed significantly better regenerative potential than the control vector-transfected hBMSCs. DNA methylation profiles showed a large difference in methylation status of genes related to osteogenesis and cell survival between hBMSCs cultured in glucose-supplemented condition and those cultured in glucose-free condition. Interestingly, METTL7A overexpression altered the methylation status of related genes to favor osteogenic differentiation and cell survival. In conclusion, it is suggested that a novel factor METTL7A enhances osteogenic differentiation and viability of hBMSCs by regulating the methylation status of genes related to osteogenesis or survival.

## Introduction

Bone can heal itself, but it is exceedingly difficult to reconstitute large bone defects induced by heavy trauma or resection of malignant tumor. In addition, if blood supply to bone defect area is deficient, bone regeneration is seriously disturbed, exemplified in osteonecrosis of the femoral head. Regenerative medicine, including the implantation of stem cells, has been studied as a novel solution to treat refractory bone defects or diseases [1, 2]. However, with impaired local vascularity, even the transplanted cells undergo rapid necrosis before differentiating into osteoblasts and regenerating bone. Without enhanced survival of implanted stem cells, regenerative therapy is inefficient [3, 4]. For example, 90% of bone marrow stem cells (BMSCS) transplanted to treat acute myocardial infarction have been reported to die within 3 days after transplantation [1]. Thus, in order to increase the effectiveness of stem cell transplantation, it is critical to improve the viability of the implanted stem cells. Most of the implanted cells die early due to exposure to hypoxia, inflammation, oxidative stress, and glucose-deficient microenvironments [5–7]. While little is known about the survival of osteogenic stem cells in the microenvironment of bone regeneration, e.g., fracture healing, several studies have shown that glucose is one of the key environmental factors necessary for osteogenic differentiation [8, 9].

In this study, assuming that the regulation of glucose may be the key to stem cell survival and osteogenic differentiation, we aimed to find the molecules that potentially replace the effect of glucose in ischemic microenvironment of stem cell transplantation for large bone defects [7, 10]. By analyzing differentially expressed genes under glucose-free conditions, which generate microenvironmental stress, and under glucose-supplemented conditions, the genetic markers that are possibly related to cell survival and osteogenic differentiation were explored. We mined methyltransferase such as 7A (METTL7A) gene, which codes for a putative enzyme that induces methylation [11–13], resulting in enhanced viability and osteogenesis of hBMSCs. The in vitro and in vivo effects of METTL7A in both glucose-free and glucose-supplemented environments as well as the underlying mechanism of action were investigated.

## Results

### Glucose is essential for osteogenic differentiation and survival of hBMSCs

**Fig. 1:**
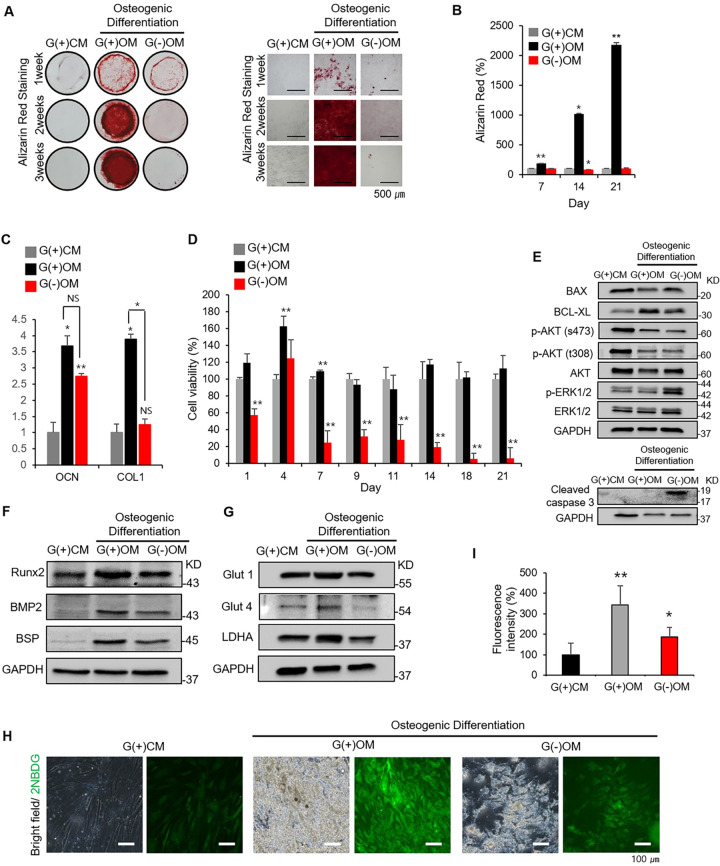
The effects of glucose on osteogenic differentiation and survival of hBMSCs. **A** Images show Alizarin Red staining of hBMSCs under various conditions, enlarged images (×100) and **B** quantification at 7, 14, and 21 days of culture. **C** Relative gene expression of osteogenic marker type I collagen (COL1) and osteocalcin (OCN) was determined by RT- on day 7 of culture. **D** Cell viability of G(+)CM, G(+)OM, and G(−)OM groups after 7, 14, and 21 days of culture. **E** Western blot of survival markers (BAX, BCL-XL, p-AKT, AKT, p-ERK, ERK, and Cleaved caspase 3) on day 7 of culture. **F** Western blot of osteogenic markers (Runx2, BMP2, and BSP), and **G** markers of glucose metabolism (Glut1, glut4, and LDHA) of hBMSCs in G(+)CM, G(+)OM, and G(−)OM groups on day 7 of culture. **H** Bright-field and fluorescent images of hBMSCs with 2NBDG tagged after 14 days of culture. **I** Quantification of fluorescent images for hBMSCs with 2NBDG tagged after 14 days of culture. Data were presented as mean ± standard deviation (SD, *N* = 3). NS: no significant difference, *p < 0.05, **p < 0.01. G(+)CM: undifferentiated hBMSCs cultured under control medium with 5.5 mM glucose; G(+)OM:osteo-induced hBMSCs cultured under osteogenic medium containing 5.5 mM glucose; G(−)OM: osteo-induced hBMSCs cultured under osteogenic medium without glucose.

Alizarin Red staining was used to confirm the effect of glucose on osteogenic differentiation. Undifferentiated hBMSCs were cultured under control medium containing 5.5 mM glucose [G(+)CM], and osteo-induced hBMSCs were cultured under osteogenic medium supplemented with 5.5 mM glucose [G(+)OM)] or under osteogenic medium without glucose [G(−)OM] for 21 days. Normally, normal blood glucose levels are defined as 4–5 mM and low glucose concentrations below 2.2 mM. Several studies also set 0–1 mM glucose as the starting level of low glucose condition [14–17]. The glucose supplementation conditions were set to the normal range of 5.5 mM and the glucose-free condition was set to 0 mM. Osteogenic differentiation was significantly enhanced in G(+)OM than in G(−)OM while almost no staining was detected under G(+)CM (Fig. 1A). When the intensity of Alizarin Red staining was analyzed graphically, the intensity was >20-fold greater in G(+)OM than G(−)OM on day 21 of culture (Fig. 1B). The gene expression of osteogenic marker type I collagen (COL1) also significantly greater in G(+)OM than in G(−)OM while that of osteocalcin (OCN) was not on day 7 of culture (Fig. 1C). Cell viability also significantly decreased by more than 70% from day 7 with G(−)OM compared with G(+)OM while no significant difference in cell viability was found between G(+)CM and G(+)OM (Fig. 1D). Western blot showed an increase in apoptosis-related protein, BAX, and a decrease in the survival-related gene, BCL-XL, under glucose-free condition. p-AKT (s473), which is associated with the survival of cells, was significantly lower in G(−)OM than in G(+)OM. Also, the expression of p-ERK that is associated with cell apoptosis was significantly higher in G(−)OM than in G(+)OM. Also, cleaved caspases 3, which plays a central role in the execution-phase of cell apoptosis, was expressed only in G(−)OM conditions, suggesting that apoptosis occurs in a glucose deficient state (Fig. 1E). The expression of proteins related to osteogenic differentiation including Runx2 and BMP2 was significantly lower with G(−)OM compared to G(+)OM. BSP expression was less with G(−)OM than with G(+)OM (Fig. 1F). Among proteins related to glucose uptake, the expression of Glut4 was significantly lower with G(−)OM than with G(+)OM. Glut1 and LDHA expression was also reduced in G(−)OM than in G(+)OM (Fig. 1G). When the glucose uptake in living cells was analyzed using 2NBDG, a fluorescent tracer used for monitoring glucose uptake into living cell, fluorescence was hardly observed in G(−)OM, whereas it was increased enormously in G(+)OM (Fig. 1H and I). These results indicate that glucose is essential for osteogenic differentiation of hBMSCs, and the absence of glucose leads to apoptosis of hBMSCs.

### Exploration of differentially expressed genes and pathways depending on glucose availability in hBMSCs

**Fig. 2:**
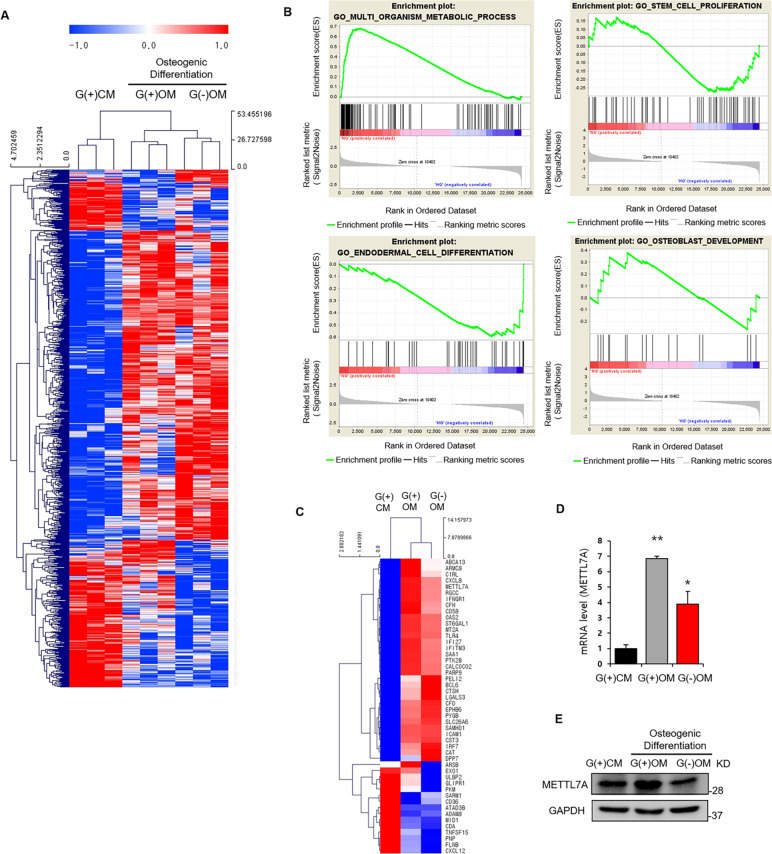
Identification of the novel gene associated with enhanced survival and osteogenic differentiation in hBMSCs. **A** RNA-seq data analysis of hBMSCs. Analysis of hierarchical clusters of genes significantly related to each sample via RNA-seq data analysis. A heatmap was generated to visualize transcriptomic differences among undifferentiated hBMSCs cultured in control medium with 5.5 mM glucose [G(+)CM], osteo-induced hBMSCs cultured in osteogenic medium containing 5.5 mM glucose [G(+)OM] or no glucose [G(−)OM] (fold-change > 2, *P* < 0.05). Red indicates upregulation, while blue indicates downregulation. **B** Pathway analysis in Functional Annotation for significant probe list was performed using DAVID for G(+)OM and G(−)OM. An analysis of difference in signaling pathways related to metabolic process, stem cell proliferation, cell differentiation, and osteoblast development. **C** A selected heatmap was generated to visualize relative transcriptomic differences among transcripts from G(+)CM, G(+)OM, and G(−)OM (fold-change > 2, *P* < 0.05). **D** Relative gene expression of METTL7A was determined by RT-qPCR, and **E** Western blot. Data were presented as mean ± standard deviation (SD, *N* = 3). NS: no significant difference, *p < 0.05, **p < 0.01. G(+)CM: undifferentiated hBMSCs cultured in control medium with 5.5 mM glucose; G(+)OM: osteo-induced hBMSCs cultured in osteogenic medium containing 5.5 mM glucose; G(−)OM: osteo-induced hBMSCs cultured in osteogenic medium without glucose.

RNA-seq analysis was performed to determine the genes and signaling pathways associated with enhanced osteogenesis and cell survival in hBMSCs. RNA sequencing data were mapped to the human reference genome, assembled per gene, and the expression of each gene was measured. Three replicates were analyzed in three different patient groups. The expression between undifferentiated BMSCs [G(+)CM] and osteogenic-differentiated hBMSCs [G(+)OM or G(−)OM] was the most distant. In addition, gene expression also varied significantly under the two conditions of osteogenic differentiation: with glucose [G(+)OM], and without glucose[G(−)OM] (Fig. 2A). Enrichment plot analysis of G(+)OM and G(−)OM showed a distinct difference in signal systems related to metabolic process, stem cell proliferation, endodermal cell differentiation, and osteoblast development between the two conditions (Fig. 2B). Next, 48 genes, which were highly differentially regulated between the two conditions according to the GO analysis, were classified, into enhanced categories (Fig. 2C). Based on the literature review, we selected 30 of those genes associated with cell survival or differentiation, and directly tested their gene and protein expression via RT-qPCR and western blot analysis. Four of these 30 genes showed a significant decrease in G(−)OM compared with G(+)OM (Fig. 2C). Recombinant proteins were treated in G(−)OM to determine the osteogenic effects in the absence of glucose. One of them, a novel factor METTL7A (methyl transferase such as 7A), revealed a significant difference between western blot (Fig. 2E) and RT-qPCR (Fig. 2D) in expression in response to glucose. The METTL7A recombinant protein showed the most prominent osteogenic effect in the absence of glucose based on Alizarin Red staining (Fig. S1).

### METTL7A knockout inhibit osteogenic differentiation and reduced cell viability in hBMSCs

**Fig. 3:**
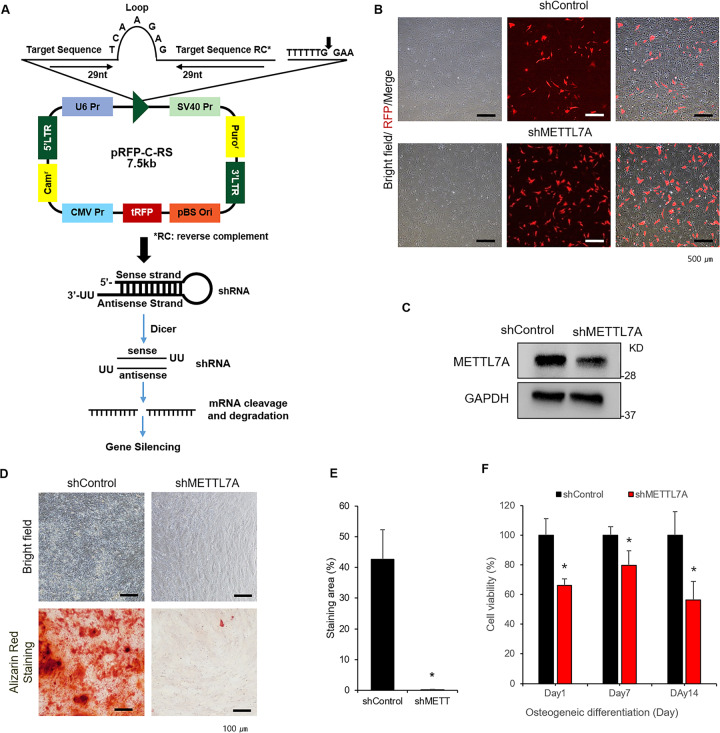
The effect of METTL7A knockdown on cell viability and osteogenic differentiation of hBMSC. **A** Construction of shMETTL7A and scramble control vector. Each vector contains RFP fluorescent genes. **B** Fluorescent, bright-field and merge images of scramble control vector- and shMETTL7A-transfected hBMSCs (RFP-tagged) after 1 day of transfection. **C** Western blot of METTL7A in scramble control vector- (shControl) and shMETTL7A-transfected hBMSCs (shMETTL7A). **D** Alizarin Red staining images and **E** quantification of shControl and shMETTL7A in osteogenic medium containing 5.5 mM glucose after 14 days. **F** Cell viability of shControl and shMETTL7A cultured under osteogenic medium containing 5.5 mM glucose on days 7, 14, and 21. Data were presented as mean ± standard deviation (SD, *N* = 3). NS: no significant difference, *p < 0.05, **p < 0.01. shControl: control vector-transfected hBMSCs; shMETTL7A:shMETTL7A-transfected hBMSCs.

Plasmid vector used to transfect shRNA into METTL7A was constructed to determine the effect of METTL7A inhibition on osteogenic differentiation and cell viability in hBMSCs. Figure 3A shows the scheme for the pRFP-C-RS plasmid vector used to generate shMETTL7A. The vector carries the shRNA sequence that inhibits METTL7A mRNA and the restriction enzyme sites that are responsive to both chloramphenicol and puromycin. pRFP-C-RS vector without the shRNA sequence was used as the control vector (Fig. 3A). The constructed vector was then introduced into hBMSCs. To confirm the transfection efficiency, bright-field and fluorescent images were confirmed by microscopy 1 day after cell transfection. RFP signal was detected in both shMETTL7A-transfected (shMETTL7A) and scramble control vector-transfected hBMSCs (shControl) (Fig. 3B). Western blotting analysis of METTLE7A demonstrated a decrease in shMETTL7A (Fig. 3C). The shMETTL7A also significantly reduced mineralized matrix formation as demonstrated by Alizarin Red staining after 14 days of incubation in osteogenic differentiation medium with 5.5 mM glucose, compared to shControl (Fig. 3D, E and S4A). Interestingly, the viability of shMETTL7A-tranfected cells was significantly reduced by ~40% on day 14 compared with that of control vector-transfected hBMSCs (Fig. 3F). These results showed that shMETTL7A was successfully transfected and inhibited METTL7A expression in hBMSCs, and that the inhibition of METTL7A by shMETTL7A reduced osteogenic differentiation and cell viability of hBMSCs in glucose-supplemented osteogenic medium.

### METTL7A overexpression enhance osteogenic differentiation and viability of hBMSCs in glucose-free conditions

**Fig. 4:**
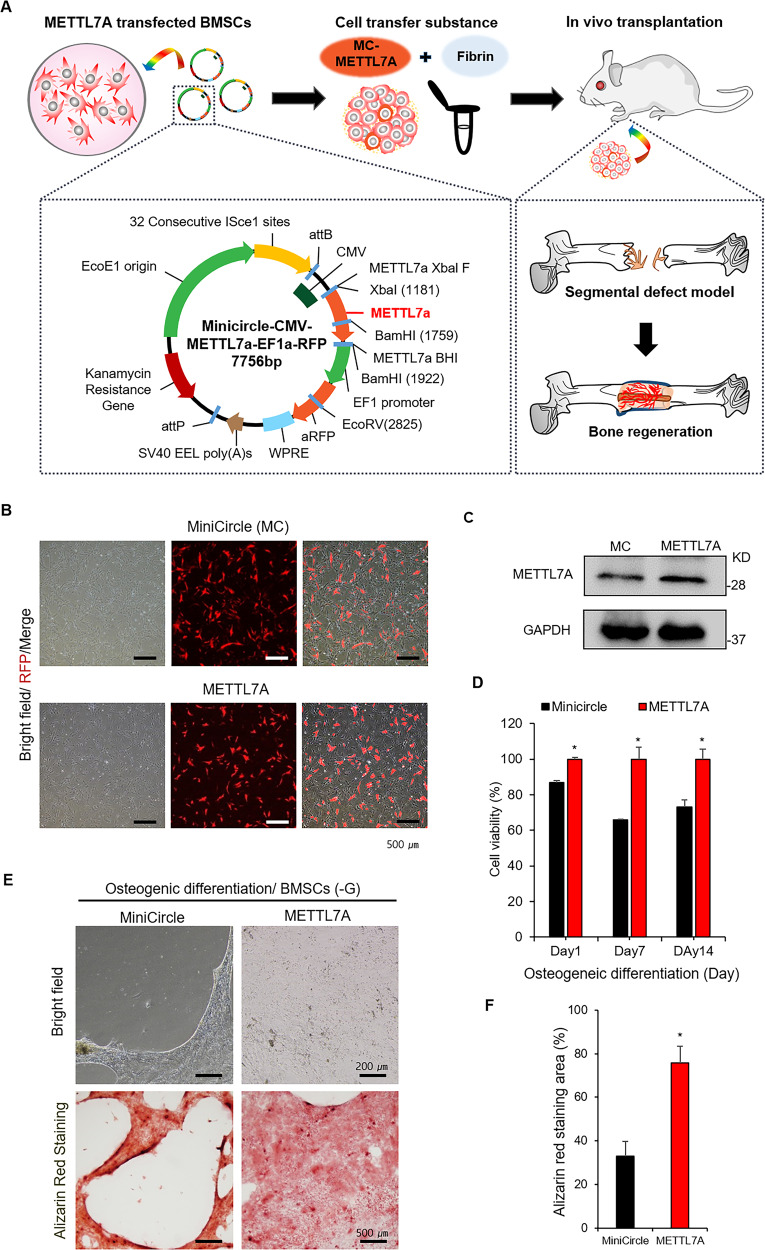
Cell viability and osteogenic differentiation of METTL7A gene-transfected hBMSCs. **A** Construction of METTL7A minicircle plasmid vector. Vector also contains RFP fluorescent genes. **B** Fluorescent, bright-field and merge images of minicircle control vector (MiniCircle)- or METTL7A-transfected hBMSCs (METTL7A; RFP-tagged). **C** Western blots of METTL7A in hBMSCs. **D** Cell viability of hBMSCs cultured in glucose-free osteogenic medium on days 7, 14, and 21. **E** Alizarin Red staining images and **F** quantification of hBMSCs cultured in glucose-free osteogenic medium after 7 days of culture. Data were presented as mean ± standard deviation (SD, *N* = 3). NS: no significant difference, *p < 0.05, **p < 0.01. hBMSCs: untransfected hBMSCs control; MC: minicircle plasmid vector (pMC)-transfected hBMSCs control; METTL7A:hMETTL7A- transfected hBMSCs.

To demonstrate the effects of METTL7A on osteogenic differentiation and cell survival of hBMSCs, the METTL7A plasmid vector was constructed. Figure 4A shows the overall scheme for the generation of METTL7A-transfected hBMSCs (METTL7A). Since the region containing bacterial skeleton is naturally degraded and disintegrated during the proliferation process, a minicircle vector containing only introduced genes was obtained as the final product. The RFP gene was also inserted into plasmids to monitor the transfection efficiency. The minicircle plasmid carrying the RFP gene was used as the control vector. The generated minicircle plasmid vector was then introduced into hBMSCs via electroporation (Fig. 4A). The fluorescent image obtained 1 day after electroporation demonstrated successful transfection of the plasmid vector as confirmed by RFP signal in both METTL7A-transfected (METTL7A) and control vector-transfected hBMSCs (MiniCircle; MC) (Fig. 4B). Western blotting confirmed the increased expression of METTL7A gene and protein in METTL7A-transfected hBMSCs (Fig. 4C). METTL7A transfection significantly enhanced the viability of hBMSCs in glucose-free condition throughout the culture period (Fig. 4D). As a next step, we tested whether METTL7A overexpression reversed the reduced osteogenic differentiation and cell viability of hBMSCs cultured under glucose-free conditions. Alizarin Red staining showed significantly enhanced formation of calcified matrix in METTL7A compared with MiniCircle after 7 days of culture in glucose-free conditions (Fig. 4E, F and S4B). Addition of human recombinant METTL7A protein led to comparable enhancement of calcified matrix formation in treated hBMSCs after 7 days of culture in glucose-free condition (Fig. S1). Also, under glucose-supplemented conditions, the METTL7A-transfected hBMSCs showed even stronger Alizarin red staining than the control vector-transfected hBMSCs after 14 days of culture (Fig. S2).

### METTL7A-transfected hBMSCs enhance new bone formation in vivo in a critical-size segmental bone defect model

**Fig. 5:**
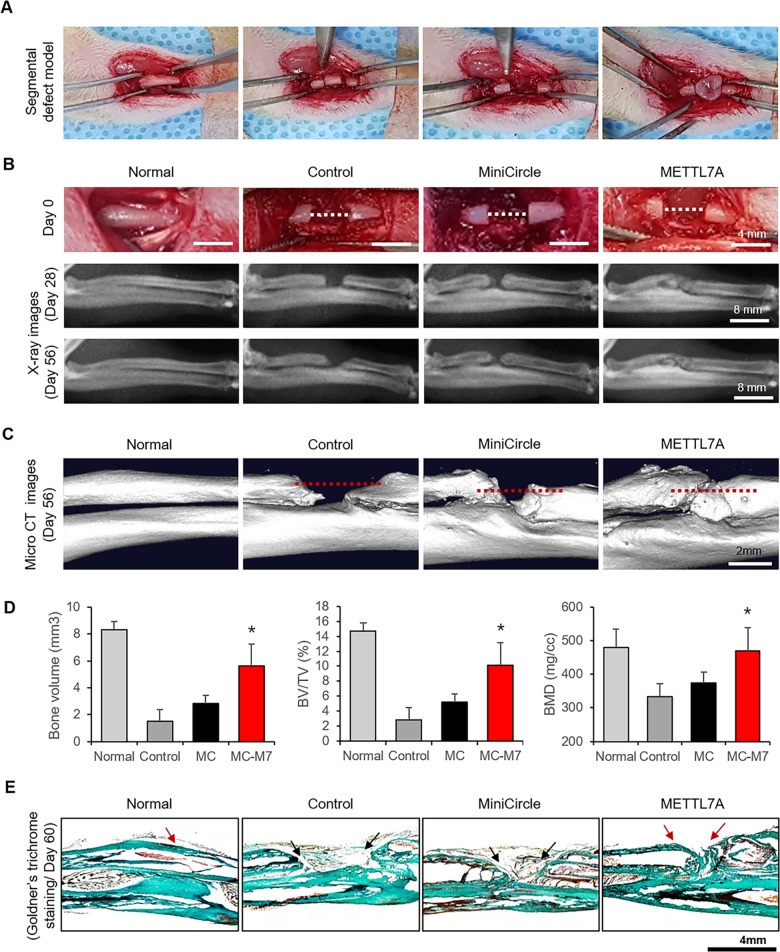
In vivo new bone formation in segmental bone defect models. **A** Creation of critical-size segmental long-bone defect in immunosuppressed rat. Length of the defects was 4 mm. hBMSCs were implanted with fibrin glue into segmental defects of rats created in the radius of right forelimb. **B** Radiographic images of segmental defects on days 28 and 56 after the implantation of hBMSCs. **C** MicroCT images of segmental long-bone defects at 56 days after cell implantation. **D** After 56 days, bone volume (BV, mm^3^), BV/TV (bone volume/total volume, %), and BMD (bone mineral density, mg/cc) were evaluated by MicroCT. **E** Goldner’s trichrome staining of healed segmental defects on day 56 post implantation of hBMSCs. Statistical processing was performed in comparison with the control group. Data were presented as mean ±standard deviation (SD, *N* = 4). NS: no significant difference, *p < 0.05, **p < 0.01. Normal: no surgery and no cell implantation; control: surgery only without cell implantation; MiniCircle (MC): minicircle control vector-transfected hBMSCs implantation; MC-METTL7A (MC-M7): METTL7A-transfected hBMSCs implantation.

The in vivo effectiveness of METTL7A-transfected hBMSCs in bone regeneration was investigated in a rat model of critical-size segmental long-bone defects. 106 METTL7A-transfected hBMSCs were implanted along with fibrin glue into 4 mm-sized segmental defects created in the radii of immunosuppressed rats (Figs. 4A and 5A; schematic diagram of the experiment process). The healing of bone defects was assessed radiographically on days 28 and 56, and via on day 56 after implantation (Fig. 5B). Bone regeneration was significantly better when implanted with METTL7A-transfected hBMSCs (METTL7A; MC-M7 group) compared with control vector-transfected hBMSCs (MiniCircle; MC group), or without cell implantation (control group) (Fig. 5B). In segmental long-bone defect model, the METTL7A group showed substantially better quality of bone regeneration, and complete healing of the segmental radial defect, while other groups had insufficient healing or gross nonunion of critical-size defects, demonstrated radiographically (Fig. 5B) and via MicroCT imaging (Fig. 5C). The bone mass (BV, mm^3^), BV/TV (bone mass/total volume, %) and bone mineral density (BMD) were re-evaluated by MicroCT. BV and BV/TV of METTL7A group was about > 2-fold higher than that of the MC group. BMD of METTL7A group was similar to that of normal bone, and also significantly greater than that of MC group (Fig. 5D). Histological findings also demonstrated that the METTL7A group had much better quality of bone regeneration than in MC group or control group (Fig. 5E). These results indicated that METTL7A overexpression enhances the in vivo regenerative potential of hBMSCs in bone healing.

### DNA methylation profiles differed significantly in hBMSCs depending on glucose availability

**Fig. 6:**
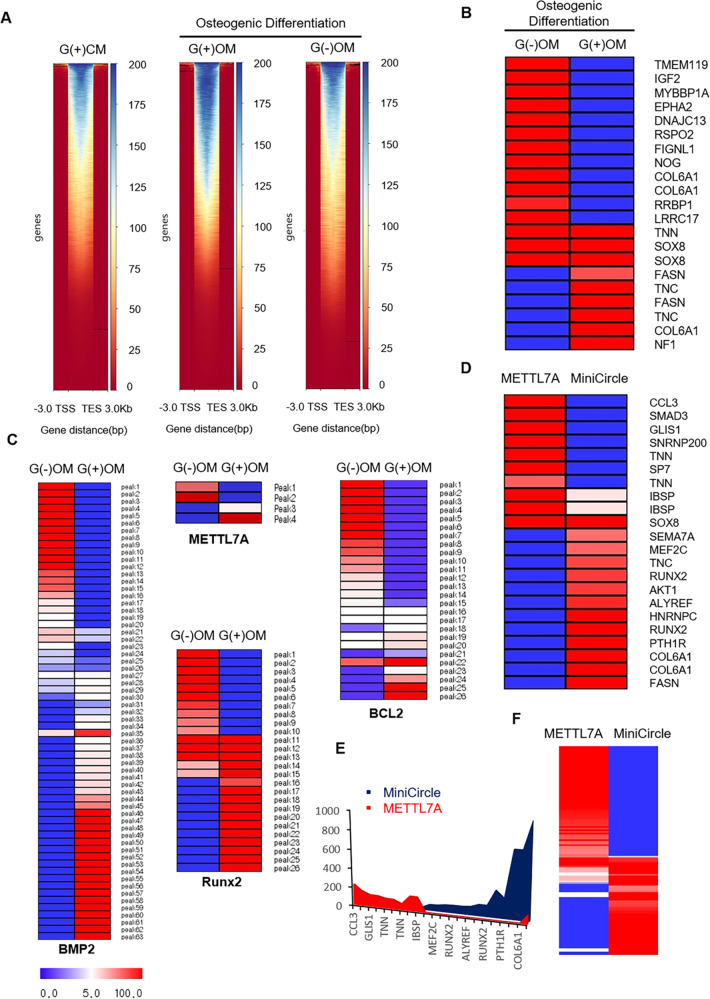
Analysis of DNA methylation profiles in hBMSCs. **A** Peak plotHeatmap using deep Tools 3.3.0 to visualize differences in methylation pattern of undifferentiated hBMSCs (BMSCs), and osteo-induced hBMSCs cultured in osteogenic medium containing 5.5 mM glucose [G(+)OM] or no glucose [G(−)OM]. Blue indicates upregulated methylation, while red indicates downregulation. **B** Heatmap of selected osteogenesis-related gene peak using MeV 4.9.0 was generated to visualize relative differences in methylation from hBMSCs cultured in osteogenic medium containing 5.5 mM glucose [G(+)OM] or no glucose [G(−)OM]. **C** Heatmap of methylation peaks for specific genes (BMP2, METTL7A, Runx2, and BCL2) in [G(−)OM] and [G(+)OM]. **D**, **E** Gene peak heatmap and graph were generated to visualize relative differences in methylation of osteogenesis-related gene between pMC-hMETTL7A- transfected hBMSCs (METTL7A) and minicircle vector (pMC)-transfected hBMSCs (MiniCircle). **F** An apoptosis-related gene peak heatmap to visualize relative differences in methylation between METTL7A and MiniCircle. hBMSCs: untransfected hBMSCs control; MC: minicircle vector (pMC)-transfected hBMSCs control; MC-METTL7A: pMC-hMETTL7A- transfected hBMSCs.

**Fig. 7:**
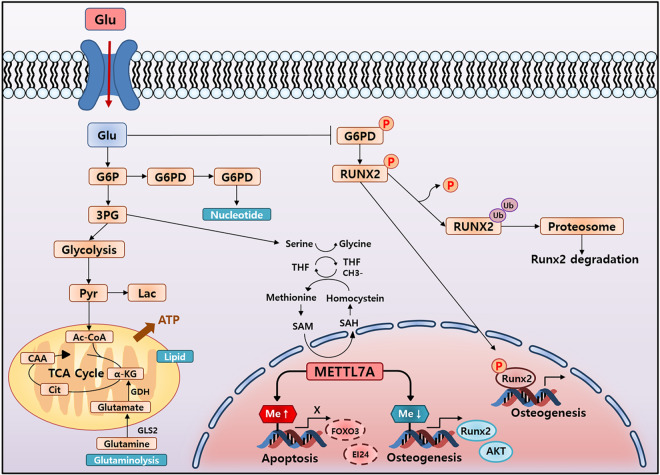
A schematic illustrating the novel relationship between genes that induce bone regeneration in human bone marrow stem cells transfected with METTL7A genes. The METTL7A gene is shown to induce bone regeneration by regulating the methylation of genes involved in osteogenic differentiation and cell survival.

DNA methylation profiles of G(+)CM, G(+)OM, and G(−)OM were investigated using deepTools 3.3.0. Dividing the “gene body length” by the size of “bin” confirms the number of bins to calculate the per-base value of each region. The average of bins for each region was determined and the heatmap was created by stacking them vertically. The heat maps are represented by transcription start site (TSS) and transcription end site (TES). The corresponding formula for each of the three comparative pairs was determined according to the scatter plot of the β-value of DNA methylation sites in the three pairs of cells. Curve fitting showed consistent DNA methylation profiles of hBMSCs under the three conditions. The methylation level in general was significantly higher in G(+)OM than in G(-)OM (Fig. 6A). A osteogenesis-related gene peak heatmap using MeV 4.9.0 was generated to visualize genes with relative differences in methylation between G(+)OM and G(−)OM, which showed significant differences in promotor methylation status of osteogenesis-related genes. Methylation of promotor regions in these genes was generally greater in G(−)OM than in G(+)OM (Fig. 6B). Figure 6C is a heatmap representing the promotor methylation peaks of specific genes selected from hBMSCs (BMP2, METTL7A, Runx2, and BCL2). BMP2 and Runx2 are associated with osteogenic differentiation while BCL2 gene is related to anti-apoptosis. The methylation peaks of those genes varied strongly between the two conditions (Fig. 6C). Gene peak heatmap and graph using MeV 4.9.0 was generated to visualize the relative differences in promotor methylation between METTL7A-transfected hBMSCs and control vector-transfected hBMSCs. After 3 days of transfection, there was a significant difference in methylation between the two types of cells (Fig. 6D and E). Methylation of osteogenic differentiation-related genes (Runx2, SOX8, COL6A1, and AKT) was greater in control vector-transfected hBMSCs than in METTL7A-transfected hBMSCs. The methylation status of apoptosis-related genes significantly differed between the two types of cells. Methylation in the promotor regions of genes related to apoptosis was greater in METTL7A-transfected hBMSCs than in control vector-transfected hBMSCs (Fig. 6F). As a result, methylation between the conditions with or without glucose at the time of osteogenic differentiation shows a marked difference, and the METTL7A gene appears to regulate this methylation. Figure 7 is a schematic diagram illustrating a new relationship between methylation and a gene that induces bone regeneration in METTL7A transfected bone marrow stem cells. The METTL7A gene is shown to induce bone regeneration by regulating the methylation of genes involved in osteogenic differentiation and cell survival.

## Discussion

While most bone defects heal by themselves or aided by conventional methods including autologous bone grafting, allografts, or bone substitutes, such lesions as large long-bone defects and osteonecrosis of femoral head are not amenable to such interventions. Therefore, regenerative medicine for bone was mainly dedicated to the possible treatment of these recalcitrant conditions. The application of stem cells, most commonly the use of mesenchymal stem cells (hBMSCs), has emerged as a potentially game-changing therapy for several medical conditions that have been refractory to conventional interventions. However, contrary to original expectations, most healing effects of implanted stem cells are attributed to paracrine effects. Implanted hBMSCs largely undergo massive cell death, failing to engraft and differentiate into host tissue [1, 2]. The massive death of grafted cells upon transplantation in the ischemic site is caused by metabolic stress due not only to hypoxia but also because of reduced supply of critical metabolic nutrients and inadequate metabolic waste removal [18].

Glucose is a primary component of metabolic homeostasis, and is a major energy source used for the synthesis of DNA, RNA, proteins, and lipids [4, 19–21]. Upon transport into the cell via the Glut family of transporters, a glucose molecule is metabolized in the cytoplasm via glycolysis to generate two pyruvate molecules, 2 ATP, and two reducing equivalents in the form of nicotinamide adenine dinucleotide (NAD). In hBMSCs survival, glucose is rapidly utilized or depleted, whereas amino acids and other required nutrients are used sparingly. Serum starvation or nutrient depletion appears to have a less notable effect on cells than glucose does [18, 20]. While the role of oxygen is pivotal to hBMSCs survival, several studies showed that hBMSCs endure sustained near-anoxia condition in the presence of exogenous glucose. Protein expression of Hif-1a and angiogenic factors was upregulated by glucose. Ectopically implanted tissue constructs supplemented with glucose exhibited 4- to 5-fold higher viability and vascularization compared with those without glucose [14]. hBMSCs survive exposure to long-term (12 days) and severe (pO_2_ < 1.5 mmHg) hypoxia in the presence of glucose. hBMSCs remained functionally viable after exposure to long-term, severe hypoxia under glucose supplementation [2]. Under low-glucose conditions, cells initiate adaptation followed by apoptosis responses using PERK/AKT and MEK1/ERK2 signaling, respectively [19]. Normally, normal blood glucose levels are defined as 4–5 mM and low glucose concentrations below 2.2 mM. Several studies also set 0–1 mM glucose as the starting level of low glucose condition [14–17]. We set the glucose-supplemented condition as the normal range of 5.5 mM and the glucose-free condition as 0 mM. In this study, similarly, the pAKT (s473) level decreased and p-ERK increased in glucose-free medium, reflecting apoptotic conditions due to metabolic stress.

While the signaling pathways involving Runx2, Wnts, and BMPs have been extensively studied in osteogenic differentiation of progenitor cells [22], the role of metabolic environment in bone regeneration has yet to be investigated. Glucose uptake induces osteoblast differentiation by suppressing the AMPK-dependent proteosomal degradation of Runx2 and promotes bone formation by inhibiting another function of AMPK. Runx2 also favors Glut1 expression, and this feedforward regulation between Runx2 and Glut1 determines the onset of osteoblast differentiation during development and the extent of bone formation throughout life [9]. In the first step of current study, we reconfirmed that glucose was essential for survival and osteogenic differentiation of hBMSCs under the osteogenic medium. Although the medium contains other nutriments including serum, a markedly reduced cell viability as well as increased expression of apoptosis-related proteins (BAX and p-ERK) and decreased expression of survival-related proteins (BCL-XL and p-AKT) suggested the need for glucose in the cell survival. Also, glucose-free conditions led to an extremely low expression of osteogenic marker proteins (Runx2, BSP) and scanty calcified matrix.

Next, we explored molecules that may replace the role of glucose in enhancing the survival and osteogenic differentiation of hBMSCs by DEG analysis. Among the 48 genes that were differentially expressed in hBMSCs with or without glucose, METTL7A was identified as a novel factor that showed significant difference in expression in response to glucose and the most prominent osteogenic effect in the absence of glucose. METTL7A, also known as AAM-B, was initially identified as a lipid droplet-associated protein in Chinese hamster ovary K2 cells via proteomic analysis [23]. It was reported as an integral membrane protein anchored into the endoplasmic reticulum membrane that recruit cellular proteins for lipid droplet formation. Interestingly, the intermediate region of METTL7A plays a putative role as S-adenosyl methionine-dependent methyltransferase [12, 24]. The methylation level of METTL7A gene was downregulated in thyroid cancer compared to normal thyroid cells [13]. It is also known to be a tumor suppressor gene with multiple editing sites at its 3′UTR [25]. However, the role of METTL7A in cell survival or osteogenic differentiation and its regulation by glucose have yet to be reported.

We tested the function of METTL7A in cell survival and osteogenesis of hBMSCs in two ways: (1) suppression of METTL7A gene expression by shRNA reduces the effect of glucose supplementation; and (2) METTL7A gene overexpression mitigates the problem of glucose deficiency. The in vitro results confirmed that METTL7A was necessary for cell survival and osteogenesis of hBMSCs, and that METTL7A overexpression can partially replace the effect of glucose. Also, the implantation of METTL7A-tranfected hBMSCs showed significantly greater in vivo osteogenic potential in critical size long-bone defects in immunocompromised rats. These results suggested that METTL7A mediates the effect of glucose in cell survival and osteogenic induction and can be used to promote stem cell-based bone regeneration.

As METTL7A acts as a methyltransferase, we finally investigated if the osteogenic induction and availability of glucose affected the methylation status of promotor regions in genome and also if METTL7A transfection altered the epigenetic status of hBMSCs. DNA methylation patterns have increasingly been implicated in transcriptional regulation and efficiency, due to advances in genome-wide DNA methylation profiling studies [26]. Current advances in transcriptional regulation by DNA methylation mostly focus on the promoter region where hypomethylated CpG islands are present with transcriptional activity, as hypermethylated CpG islands generally result in gene repression [27–29]. Environmental factors induce specific phenotypic changes in genomic DNA through epigenetic modification [30]. Although DNA methylation is known to be the most important epigenetic regulator of mammalian development [31], studies involving altered methylation status due to metabolic stress in osteogenic differentiation of progenitor cells have yet to be reported.

Our results from DNA methylation profiling of promotor regions suggested large differences in methylation status of osteogenesis (BMP2, Runx2) or survival (BCL2)-related genes in hBMSCs depending on glucose availability. METTL7A transfection appears to decrease the methylation of osteoblast differentiation-related genes while increasing that of apoptosis-related genes. Therefore, it is possible that glucose supplementation increased intracellular expression of METTL7A, which in turn leads to altered methylation status of related genes to favor osteogenic differentiation and cell survival. To the best of our knowledge, this is the first reported evidence supporting the role of METTL7A in cell survival and osteogenic differentiation by changing the promotor methylation status of related genes. However, it is not known from the results of this study and awaits further investigation how the increased glucose level leads to increased METTL7A gene expression and how METTL7A differentially methylate certain gene promotors.

## Materials and methods

### Cell culture

Human bone marrow stem cells (hBMSCs) were isolated from bone marrow of seven patients (mean age: 74 years; range: 60–87 years). Informed consent was obtained from all donors. All experiments were performed in accordance with the relevant guidelines and regulations. The collection of human samples was approved by the Institutional Review Board at Dongguk University Ilsan Hospital (IRB file no. DUIH 2012-01-034). Bone marrow obtained from the human body was diluted with Dulbecco’s phosphate-buffered saline (DPBS: Welgene, Cat. LB 001-02, Republic of Korea) and transferred to a conical tube, supplemented with Lymphoprep (Alere Technologies AS, Cat. 1114544, Norway) in a 1:1.25 ratio. The oil layer was removed by centrifuging at 2000 rpm, brake 0, 30 min, and the cell layer was diluted 1:2 with DPBS after collecting separately in a new tube. The hBMSCs pellet was obtained by centrifuging at 1500 rpm for 8 min from diluted cell layer. The hBMSCs were incubated at 1 × 10^7^ cells per 100 mm dish in αMEM (Gibco, Cat. 12571-063, Grand Island, NY, USA) supplemented with 10% FBS (Gibco, Cat. 16000-044, Grand Island, NY, USA) and 1% penicillin–streptomycin 100X (Welgene, Cat. LS 202-02, Republic of Korea.) at 37 °C under 5% CO_2_. It was tested for mycoplasma contamination and was confirmed to be free from contamination.

To further investigate osteogenic differentiation, 2.5 × 10^5^ cells were placed in each well of a 6-well plate. Osteogenic differentiation was induced by replacing the osteogenic medium (OM: DMEM with 10% FBS, 1% penicillin–streptomycin, 10 mM glycerolphosphate, 50 nM L-ascorbic acid-2-phosphate, 100 nM dexamethasone) at 37 °C under 5% CO_2_. DMEM with low glucose (Welgene, Cat. LM 001-06, Republic of Korea) was used to make 5.5 mM glucose-supplemented osteogenic medium, and DMEM without glucose (Gibco, Cat. 11966-025, USA) to make glucose-free osteogenic medium (OM). The maintenance medium for hBMSCs also contained 5.5 mM glucose, and was used as control medium (CM), designated as glucose-supplemented control medium.

### Construction of MiniCircle Vector

pMC-CMV-MCS (MN501A-1), a vector to produce MiniCircle (MC), was purchased from Systemic Biosciences (SBI, Palo Alto, CA, USA) and used as a basic framework to prepare the plasmid vector. Sequences capable of expressing METTL7A genes were inserted downstream of the CMV promoter using the BamHI and NheI restriction sites of the parent plasmid (pMC) for gene cloning. METTL7A gene was purchased as MGC clones provided by Invitrogen, and amplified by PCR. The primers for PCR of each gene are as follows (NM_014033): (METTL7A forward: CCTTCTGAGCAATGGAGCTT, METTL7A reverse: GGTGGCTGACGTCTGTAATCA). The genes obtained after PCR were ligated to pGEMT- Easy vector for full gene sequencing (Fig. S3A and B). METTL7A genes with exactly matching gene sequences were inserted into pMC.

### Transfection of METTL7A gene to hBMSCs by electroporation

The supernatant was removed by centrifuging hBMSCs at 1000 rpm for 3 min. A suspension of 10^6^ cells supplemented with 100 µL of R buffer (Neon™ Transfection System, MPK10096, CA, USA) and 10 μg METTL7A-expressing nonviral MiniCircle vectors (MC-METTL7A) was obtained. To confirm the transfection efficiency, MC-METTL7A was constructed to include red fluorescent protein (RFP). Using electroporation devices (Neon™ Transfection System, MPK5000, CA, USA), MC-METTL7A was transfected at 1050 V, 30 ms, and 2 pulses. hBMSCs were cultured in αMEM (Gibco, Cat. 12571-063, Grand Island, NY, USA) without antibiotics. Transfection efficiency was confirmed by counting the fluorescent cells.

### Preparation and transfection of shRNA vector

The METTL7A Human shRNA Plasmid Kit (Origene, Cat. TF311498, MD, USA) and pRFP-C-RS Vector (Origene, Cat. TR30014, MD, USA) was used. The plasmid contains the sequence that inhibits METTL7A mRNA and the restriction enzyme sites that are responsive to chloramphenicol and puromycin. To confirm the transfection efficiency, the RFP gene was inserted into the vector. The pRFP-C-RS shRNA vector was used as the control. Transfection was performed using 2.5 µg of TurboFectin transfection reagent (Origene, Cat. TF81001, MD, USA) mixed with 2 × 10^5^ cells according to the manufacturer’s instructions. Transfected cells were cultured for 2 days at 37 °C under an atmosphere of 5% CO_2_ and selected with 2 µg/mL puromycin for 3 days. The transfection efficiency was confirmed through fluorescence expression.

### RT-qPCR analysis

Total RNA was isolated from hBMSCs using Direct-zol™ RNA MiniPrep Plus (200 preps, Zymo Research, Cat. R2072, Irwin, CA, USA). The cDNA was synthesized using RT-qPCR with Maxime RT PreMix (Oligo dT primer: iNtRON, Cat. 25081, Republic of Korea) in a SimpliAmp Thermal Cycler (Applied Biosystems, Foster City, CA, USA). The primers used are shown in Table 1.

**Table 1:** The primers used in RT-qPCR.

Primer	Sequence (5’→3’)
*Methyltransferase Like 7A* forward	*GTGCAACCTGACCAGAGAGA*
*Methyltransferase Like 7A* reverse	*GTGCTGCAGCTTCAGCTTAG*
*Osteocalcin* forward	*TCAGCCAACTCGTCACAGTC*
*Osteocalcin* reverse	*GGGGCTACCTGTATCAATGG*
*collagen type I alpha 1 chain* forward	*CCAGTTCTTCATTGCATTGC*
*collagen type I alpha 1 chain* reverse	*AACCCGAGGTATGCTTGATCT*

### Alizarin Red staining

To evaluate the osteogenic differentiation of hBMSCs from the calcified matrix, cells were stained with Alizarin Red S (Sigma-Aldrich, Cat. A5533-25G, Saint Louis, MO, USA). After osteogenic differentiation for 7 days, the cells were fixed with 4% paraformaldehyde, then stained with 2% Alizarin Red S and dried. Alizarin Red staining area (%) was measured using the ImageJ program (NIH).

### Cell viability analysis

Cell viability analysis is used to determine the degree to which tetrazolium salt is reduced by mitochondrial NADH dehydrogenase in cells and converted into a colored formazan. In this assay, 1 × 10^4^ hBMSCs were dispensed to each well of 96-well plate, suspended in 100 µL of each culture medium, and incubated at 37 °C under an atmosphere of 5% CO_2_ for 24 h. Analysis was performed using the EZ-Cytox kit (DoGEN, Cat. EZ-1000, Republic of Korea) as described by the manufacturer’s instructions. The reagent contained in the above kit was added to the medium in the ratio of 1:10 in micro-level quantities. After 1 h, the optical density was measured at 450 nm wavelength with a VersaMax Microplate Reader (Molecular Devices, Sunnyvale, CA, USA).

### Western blotting analysis

Cells were dissolved in a radioimmunoprecipitation assay buffer (Thermo Scientific, Cat. 89900, Waltham, MA, USA) containing Halt™ Protease & Phosphatase Inhibitor Cocktail (Thermo Scientific, Waltham, MA, USA). Protein concentration was measured using a bicinchoninic acid assay, and the dissolved proteins were separated by SDS-polyacrylamide gel electrophoresis for 2 h, and transferred to a nitrocellulose membrane (Whatman®, Cat. E06-07-111, UK), followed by blocking with 5% skimmed milk in 1× Tris-buffered saline with 2% Tween-20. Thereafter, the cells were reacted overnight at 4 °C with the primary antibodies (1:1000, Table 2), followed by the secondary antibody (1:1000) [anti-rabbit IgG horseradish peroxidase (HRP)-linked antibody, #7074, Cell Signaling /anti-mouse IgG HRP-linked antibody, #7076, Cell Signaling] at room temperature (RT) for 2 h. Antibody–antigen complexes were detected using SuperSignal™ West Femto Maximum Sensitivity Substrate (Thermo Scientific, Cat. 34095, Waltham, MA, USA), and signal intensities were measured using the ChemiDoc™ XRS + Imaging System (Bio-Rad, Hercules, CA, USA).

**Table 2:** Primary antibodies used in western blotting analysis.

Target	Antibody	Company	Catalog No.
*METTL7A*	Anti-METTL7A antibody	Abcam	Ab79207
*BSP*	Anti-Bone Sialoprotein antibody	Abcam	Ab33022
*Runx2*	Mouse-Runx2 antibody	Abcam	Ab76956
*BMP2*	Anti-BMP2 antibody	Abcam	Ab14933
*Glut1*	Glut1 Antibody (A-4)	SCBT	sc-377228
*Glut4*	Anti-Glucose Transporter GLUT4 antibody	Abcam	Ab216661
*LDHA*	LDHA (C4B5) Rabbit mAb	Cell Signaling	C4B5
*BAX*	Bax Antibody	Cell Signaling	#2772S
*BCL-XL*	Bcl-xL (54H6) Rabbit mAb	Cell Signaling	#2764S
*p-AKT(s473)*	Phospho-Akt (Ser473) (D9E) XP® Rabbit mAb	Cell Signaling	#4060S
*p-AKT(t308)*	Phospho-Akt (Thr308) (D25E6) XP® Rabbit mAb	Cell Signaling	#13038S
*AKT*	Akt (pan) (C67E7) Rabbit mAb	Cell Signaling	#4691S
*p-ERK*	Phospho-p44/42 MAPK (Erk1/2) (Thr202/Tyr204) Antibody	Cell Signaling	#9101S
*ERK*	p44/42 MAPK (Erk1/2) (137F5) Rabbit mAb	Cell Signaling	#4695
*Cleaved caspase3*	Cleaved Caspase-3 (Asp175) (5A1E) Rabbit mAb	Cell Signaling	#9664
*GAPDH*	Rabbit-GAPDH antibody	BETHYL	A300-641A-M

### Critical-sized segmental long-bone defect in rats

An 11-week-old male Sprague-Dawley (SD) rat was used in the bone defect model. Five animals were assigned to each of the following four groups randomly: normal (no surgery, no cell implantation), control (defects only without cell implantation), MiniCircle (pMC control vector-transfected hBMSCs implantation), and MC-METTL7A (METTL7A-transfected hBMSCs implantation). All animal management procedures, anesthesia, and surgeries were performed in compliance with the ARRIVE guidelines and the protocol of Dongguk University Institutional Animal Care and Use Committee. Segmental long-bone defects were created by drilling to a depth of 4 mm in the proximal radius of SD rats. 1 × 10^6^ METTL7A-transfected hBMSC or control vector-transfected hBMSCs mixed with 20 µL fibrin/thrombin (Green Cross, Republic of Korea) were implanted into the segmental long-bone defects. The rats were injected with immunosuppressive agents (CIPOL; Chong Kun Dang, Republic of Korea) daily, 2.5 mg/300 µL in the first week and 0.83 mg/100 µL from 2 to 8 weeks. After 8 weeks, bone regeneration was confirmed by visual observation, X-ray, and MicroCT. Bone volume (BV, mm^3^), BV/TV (bone volume/total volume, %), and BMD (bone mineral density, mg/cc) were evaluated by MicroCT.

### Goldner’s trichrome staining

The segmental bone tissue was frozen into blocks using Tissue-Tek O.C.T. Compound (Sakura Finetek, 4583, Torrance, CA, USA). Using a freeze-cutting machine (Leica CM1950 Cryostat; Leica Microsystems, Wetzlar, Germany), tissue blocks were sectioned to 20 µm in thickness. Tissue slices were washed with distilled water and treated with a 1:1 mixture of hematoxylin solution II (Merck Millipore, Cat. HX384856, Billerica, MA, USA) and III (Merck Millipore, Cat. HX303261, Billerica, MA, USA). After washing with flowing water, Goldner I, II, and III solutions (Carl Roth, Cat. Art.-Nr. 3469.1, 3470.1, 3473.1, Karlsruhe, Germany) were used sequentially for 5 min each. Finally, after washing with distilled water, the stained tissue slice was mounted, and observed under a microscope.

### Glucose uptake assay

Glucose uptake in living cells was observed using 2-NBDG. hBMSCs were seeded at 1.25 × 10^5^ cells per well in 12-well plates and cultured in different media at 37 °C and 5% CO_2_. Cells were washed three times with DPBS and treated with 60 μM 2-NBDG (Biogems, Cat. 1860768, CA, USA). After 30 min at 37 °C and 5% CO_2_, glucose uptake was evaluated based on fluorescence expression.

### RNA-seq assay

Total RNA was extracted from the samples using the TRIzol reagent (Invitrogen, Carlsbad, CA, USA). The concentration and purity were determined in terms of optical density at A260 and A260/A280, respectively, using a spectrophotometer (Bio-Tek Instruments). RNA sequencing library of each sample was prepared using the TruSeq RNA Library Prep Kit (Illumina, San Diego, California, USA). RNA-seq experiments were performed in hBMSCs cultured under different conditions each. RNA-seq analysis was performed using the Illumina HiSeq 2000 system. Gene expression was normalized to RPKM/FPKM (reads of paired end fragments per kb of exon model per million mapped reads/fragment per kb of transcript per million mapped reads). The quality of the sequencing reads generated in the RNA-seq experiment was confirmed using the Excel-based Differentially Expressed Gene Analysis (ExDEGA) tool. A heatmap was generated to visualize transcriptomic differences between hBMSCs cultured under control medium containing 5.5 mM glucose [G(+)CM], osteo-induced hBMSCs were cultured under osteogenic medium supplemented with 5.5 mM glucose [G(+)OM)] and under osteogenic medium without glucose [G(−)OM].

### MBD-seq assay

MBD-seq experiments were performed in groups of hBMSCs [undifferentiated hBMSCs with 5.5 mM glucose; G(+)CM], (+) glucose [osteogenic differentiation hBMSCs with 5.5 mM glucose; G(+)OM] and (−) glucose [osteogenic differentiation of hBMSCs without glucose; G(−)OM]. For each sample, DNA fragments of less than 1000 bp were prepared. Analysis was performed using the Methylated DNA Enrichment Kit (New England Biolabs., Cat. E2600S, USA) according to the manufacturer’s instructions. MBD2-Fc protein and Protein A Magnetic Beads were suspended. Methylated CpG DNA was captured by adding 1 μg of fragmented sample DNA, DNase-free water and 5× Bind/Wash Buffer. After incubating the DNA and MBD2a-Fc/Protein A Magnetic Beads, the supernatant was removed to wash off unbound DNA. The magnetic bead pellet sample and DNase-free water were mixed and incubated in a heat block at 65 °C for 15 min to obtain a supernatant, which contains enriched methyl CpG containing DNA. Cluster generation and 200 bp paired-end sequencing were performed on an Illumina HiSeq2500 instrument (Illumina, San Diego, CA, USA). We performed peak plotHeatmap using deepTools 3.3.0. This tool creates a heatmap for scores associated with genomic regions. We performed gene peakHeatmap using MeV 4.9.0. The value input into the analysis represents the raw read value of each peak.

### Statistical analysis

The investigators were blinded to the group allocation during the experiments of the study. The in vitro experiments were performed in triplicate, with similar results obtained. Data were presented as mean ± standard deviation (SD). In this study, the significance of the differences was determined using the Student *T*-test to analyze multiple groups. *P* values less than 0.05 were considered to indicate statistical significance.

## Conclusion

We mined a novel factor METTL7A based on glucose metabolic regulation and identified previously unknown roles of METTL7A in enhancing cell survival and inducing osteogenic differentiation of hBMSCs. It is expected that METTL7A overexpression possibly protects implanted cells against metabolic stress due to glucose deficiency. Accordingly, this factor can be used in stem-cell based bone tissue engineering in refractory conditions including large bone defects or osteonecrosis of femoral head.

## Supplementary information

Supplement Figure 4

Supplement Figure legend

Supplement Figure 1

Supplement Figure 2

Supplement Figure 3
