# Supplementary figures and images for: Methyltransferase-like protein 7A (METTL7A) promotes cell survival and osteogenic differentiation under metabolic stress
Source: Cell Death Discov. 2021 Jun 30;7:154. doi: 10.1038/s41420-021-00555-4 (PMC8257615; doi:10.1038/s41420-021-00555-4)

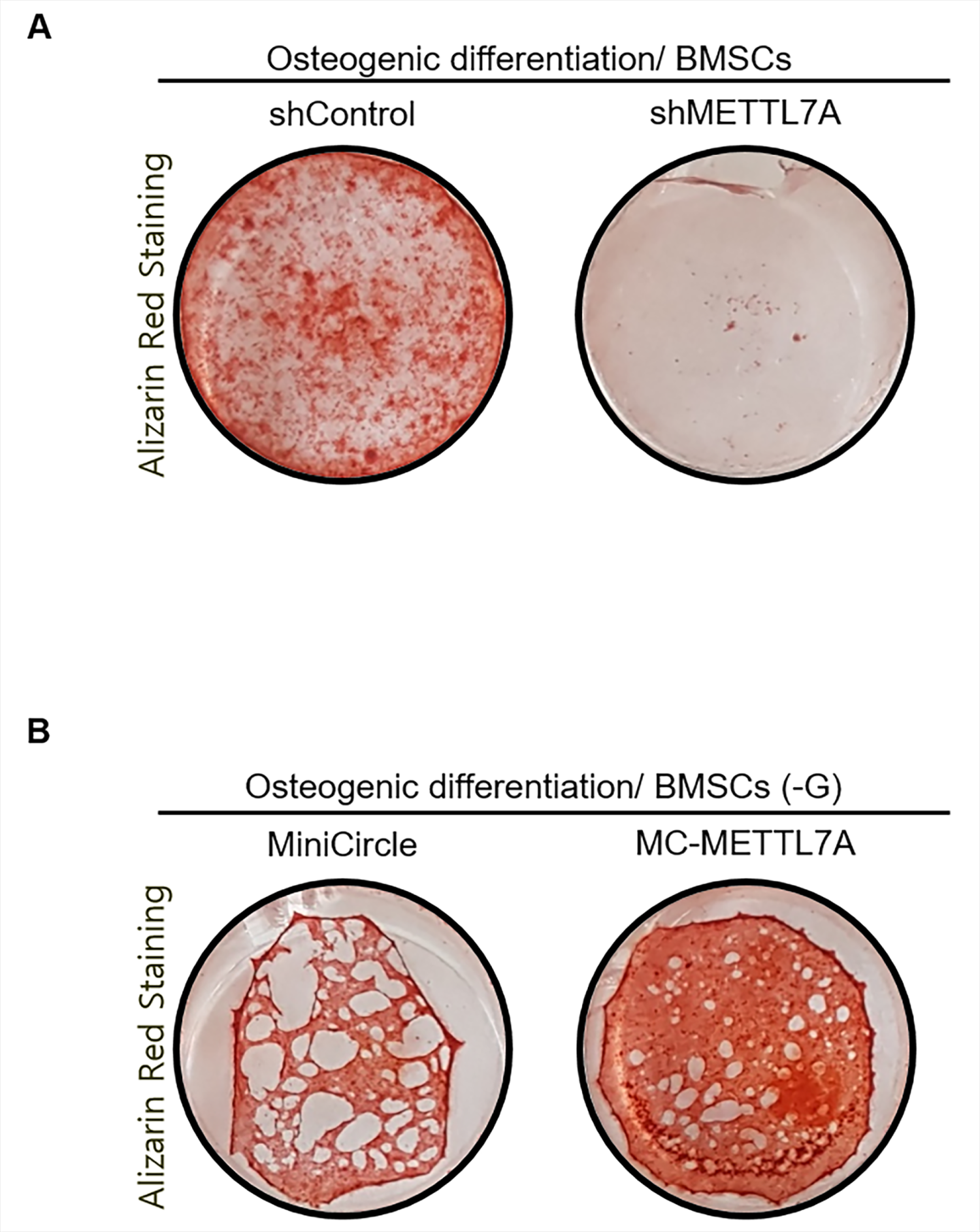

Supplement: Supplementary file 1 — Supplement Figure 4 [file 41420_2021_555_MOESM1_ESM.png]

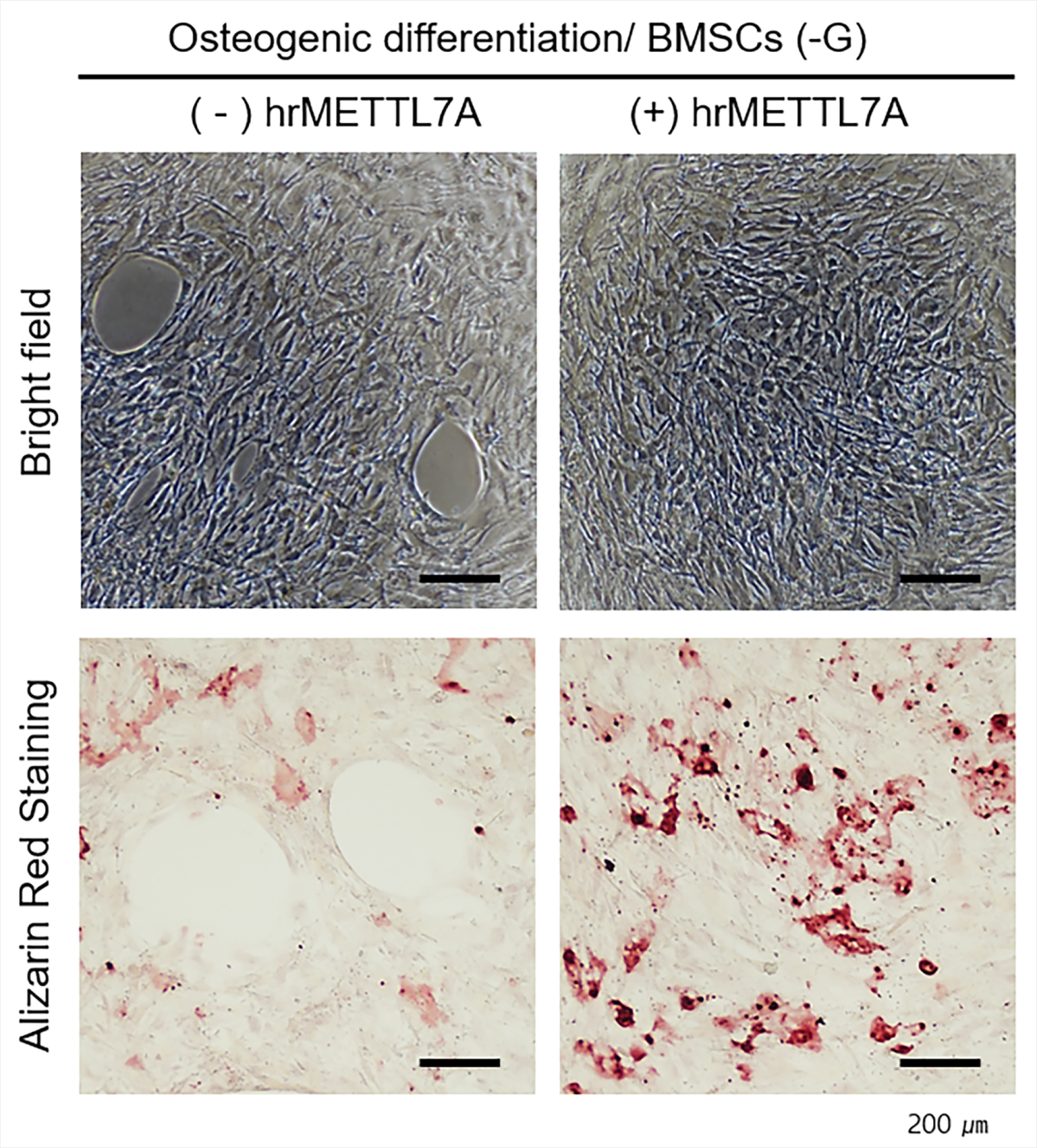

Supplement: Supplementary file 3 — Supplement Figure 1 [file 41420_2021_555_MOESM3_ESM.png]

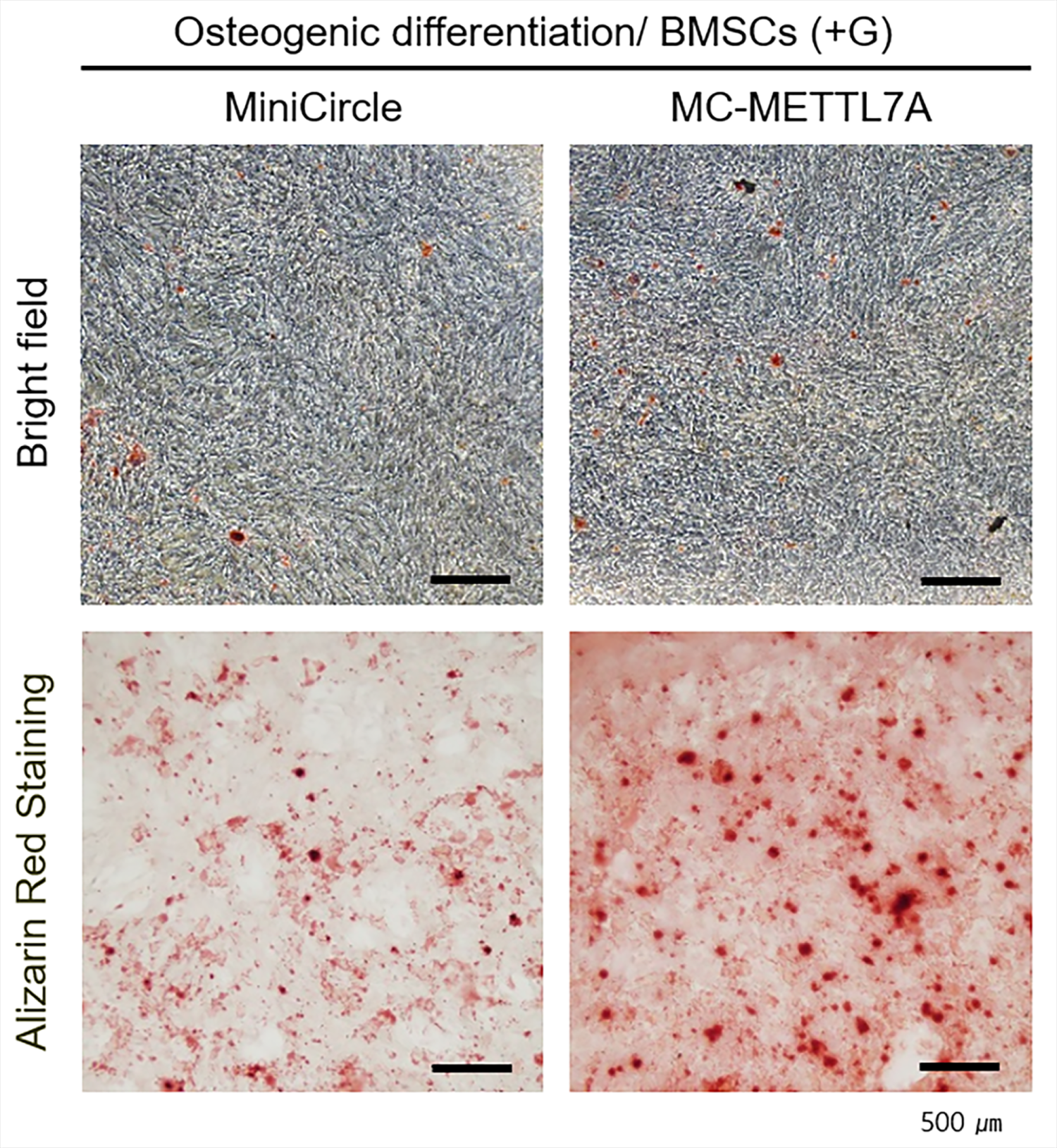

Supplement: Supplementary file 4 — Supplement Figure 2 [file 41420_2021_555_MOESM4_ESM.png]

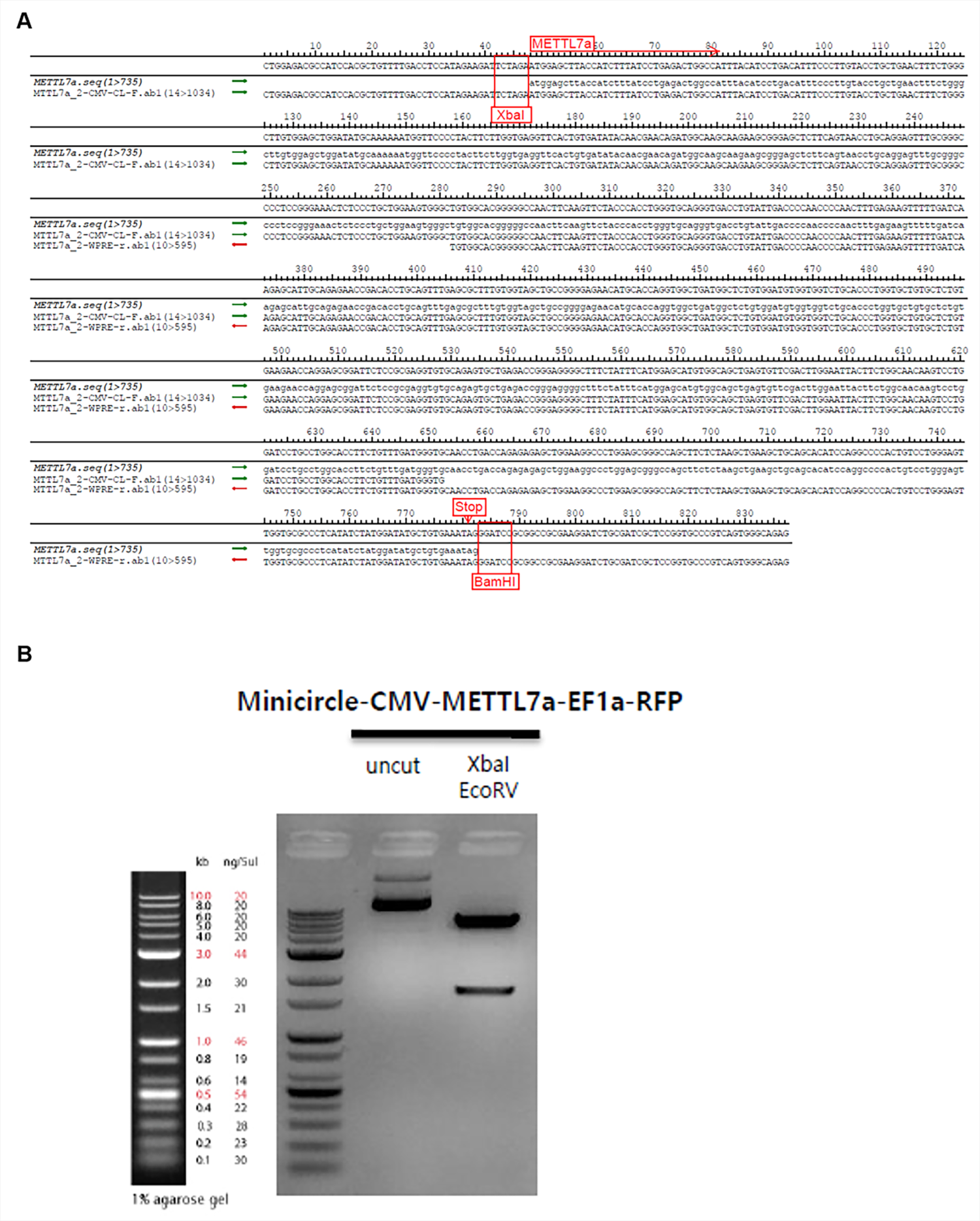

Supplement: Supplementary file 5 — Supplement Figure 3 [file 41420_2021_555_MOESM5_ESM.png]
