# Supplementary material for: Methyltransferase-like protein 7A (METTL7A) promotes cell survival and osteogenic differentiation under metabolic stress
Source: Cell Death Discov. 2021 Jun 30;7:154. doi: 10.1038/s41420-021-00555-4 (PMC8257615; doi:10.1038/s41420-021-00555-4)
Supplement: Supplementary file 2 — Supplement Figure legend [file 41420_2021_555_MOESM2_ESM.docx]

**SUPPLEMENTARY FIGURE LEGENDS**

**Fig S1. Osteogenic effect of the *METTL7A* recombinant protein in the absence of glucose.** Alizarin Red staining images of hBMSCs cultured in glucose-free osteogenic medium with METTL7A recobminant protein after 7 days of culture.

**Fig S2. Osteogenic effect of the *METTL7A*-transfected hBMSCs in glucose condition.** Alizarin Red staining images of hBMSCs cultured in glucose osteogenic medium with minicircle control vector (MiniCircle)- or METTL7A-transfected hBMSCs after 14 days of culture.

**Fig S3.** (A) The genes obtained after PCR were ligated to *pGEMT*- Easy vector for full gene sequencing. (B) METTL7A genes with exactly matching gene were inserted into minicirle vector*.* The RFP gene was also inserted into Minicircle-CMV-METTL7A-EF1a plasmids.

**Fig S4.** (A) Alizarin Red staining after 14 days of incubation in osteogenic differentiation medium with 5.5 mM glucose for *shControl* and *shMETTL7A*. **(B)** Alizarin Red staining of METTL7A compared with MiniCircle after 7 days of culture in glucose-free conditions. MiniCircle: minicircle plasmid vector (pMC)-transfected hBMSCs control; METTL7A:hMETTL7A- transfected hBMSCs. shControl: control vector-transfected hBMSCs; shMETTL7A:shMETTL7A-transfected hBMSCs.
